# New MADS-Box Gene in Fern: Cloning and Expression Analysis of *DfMADS1* from *Dryopteris fragrans*

**DOI:** 10.1371/journal.pone.0086349

**Published:** 2014-01-22

**Authors:** Qingyang Huang, Wenhua Li, Ruifeng Fan, Ying Chang

**Affiliations:** 1 Laboratory of Plant Research, College of Life sciences, Northeast Agricultural University, Harbin, Heilongjiang Province, P. R. China; 2 Institute of Natural Resources and Ecology, Heilongjiang Academy of Science, Harbin, Heilongjiang Province, P. R. China; 3 Key Laboratory of Chinese Materia Medica (Ministry of Education), Heilongjiang University of Chinese Medicine, Harbin, Heilongjiang Province, P. R. China; University of South Florida College of Medicine, United States of America

## Abstract

*MADS* genes encode a family of transcription factors, some of which control the identities of floral organs in flowering plants. Most of the *MADS-box* genes in fern have been cloned and analyzed in model plants, such as *Ceratopteris richardii* and *Ceratopteris pteridoides.* In this study, a new *MADS-box* gene, *DfMADS1*(GU385475), was cloned from *Dryopteris fragrans* (L.) Schott to better understand the role of MADS genes in the evolution of floral organs. The full-length *DfMADS*1 cDNA was 973 bp in length with a 75bp 5′-UTR and a 169bp 3′-UTR. The DfMADS1 protein was predicted to contain a typical MIKC-type domain structure consisting of a MADS domain, a short I region, a K domain, and a C-terminal region. The DfMADS1 protein showed high homology with MADS box proteins from other ferns. Phylogenetic analysis revealed that *DfMADS1* belongs to the CRM1-like subfamily. RT-PCR analysis indicated that *DfMADS*1 is expressed in both the gametophytes and the sporophytes of *D. fragrans*.

## Introduction

*MADS* genes encode a family of transcription factors that play important roles in signal transduction and developmental control in plants, animals, and fungi [1]–[3]. In *Arabidopsi*s and other higher flowering plants, studies have shown that *MADS-box* genes are key regulators of flower development [4].

MADS domain proteins show a modular organization comprising a MADS (M) domain, an intervening (I) region, a keratin-like (K) domain, and a C-terminal (C) domain, also called the MIKC-type MADS-box [5]. The M domain is by far the most highly conserved region of the proteins [6], featuring a highly conserved 60 aa sequence that functions in DNA binding, dimerization, and accessory-factor interactions [7], [8]. An additional conserved domain composed of 70 aa residues, called the K box, potentially forms amphipathic helices involved in protein–protein interactions [9].

The function and evolution of *MIKC MADS-box* genes have been intensively studied in a large number of plant species [10]–[12]. The *MADS-box* gene family diverges into two broad clades, types I and II, which existed before the divergence of plants, animals, and fungi [13]. In land plants, type II *MADS-box* genes further diverge into two groups, namely MIKC^C^ and MIKC* [14].

Fifteen different *MADS-box* genes have been cloned in *Ceratopteris richardii* and *Ceratopteris pteridoides*, and the majority of these genes have high sequence similarity with MIKC^C^-type *MADS-box* genes in typical seed plants [15]. These findings suggest that ferns and seed plants may have a common ancestor.

Phylogenetic reconstruction shows three divergent *MADS-box* gene groups in *Ceratopteris* incorporated into the gene clade of seed plants [16]; these groups, however, are considerably smaller than the number of *MADS* gene groups in seed plants. These findings suggest that at least two different *MADS-box* gene types of the MIKC class exist in the common ancestor of fern and seed plants.

The fern genus *Dryopteris* (Dryopteridaceae) is among the most common and species-rich fern genera in temperate forests in the northern hemisphere, comprising 225 to 300 species worldwide. *Dryopteris fragrans* is located in Filicales in the taxonomic system and belongs to the *Dryopteris* genus of the Dryopteris family [17]. This fern is called fragrant cliff fern, fragrant wood fern, or Dryoptère odorante in other countries. According to molecular circumscription studies, *D. fragrans* belongs to the single Fragrantes clade and is a sister to the rest of *Dryopteris* [18]. Thus, studies on the *MADS-box* gene of *D. fragrans* are necessary and significant. In the current study, we cloned a MADS gene from *D. fragrans* and analyzed its expression. This study lays the foundation for future research on the function of *MADS-box* genes in ferns.

## Materials and Methods

### Plant Materials and Culture Conditions

*D. fragrans* and its spores, which developed in one year, were collected from Wudalianchi, Heilongjiang, China (126°07′07″N, 48°42′38″E). No specific permissions were required because the sample location is an experimental area that allows researchers do their work so long as their activities do not break the law. *D. fragrans* is not an endangered or protected species in China.

The wild plant was transplanted into a botany laboratory in the Northeast Agricultural University. New unfolded leaves were collected for RNA extraction. The spores were cultured in solid culture medium, and different developmental stages of sterile seedlings were transplanted to the soil under a 10 h/14 h photoperiod at 25/22°C day/night with 150–180 µmol·m^−2^·fs^−1^ light intensity [19].

Various tissues of *D. fragrans,* including leaf, petiole, root, and developing tissues, at different stages of growth (gametophyte, young prothallus, aborted prothallus, young leaf, young sporangium, mature sporangium) were collected, frozen in liquid nitrogen, and stored at −80°C for RNA extraction.

### Gene Cloning

Total RNA was extracted from leaves using an RNA plant kit (Qiagen, Germany). For 3′ full RACE, cDNA was synthesized from mRNA with the Oligo dT-Adaptor primer using an RT-PCR kit (version 3.0; TaKara, Japan). The 3′-RACE degenerate primer was designed according to the conserved sequence of *MADS*-box genes in pteridophytes. The 3′-RACE primer used was 5′-AAG AAA GCN YAC GAK CTR TC-3′ (where R = A or G; I = inosine; Y = Cor T; N = A, C, G, or T) and the 3′-RACE specific primer was provided by the RT-PCR kit (TaKara). For 5′full RACE, the nest PCR primer was carried out using 5′RACE System v2.0 (Invitrogen), and the cDNA was synthesized from mRNA using the primer 1-RT 5′-GCCAATGAGGTTGCTCTGTA-3′. The primers used for the first round of PCR reaction were DfMADS 1-1R 5′-CGTTATCGCTGTGATCGTCTG-3 and APP. The second round of PCR was performed using AUAP and DfMADS1-2R 5′-CGGTTGCTATCAGCGTATCG-3′. Approximately 1–2 µL of the first-round PCR products was used as a template for the second round of PCR. APP and AUAP were nested universal primers provided in the kit.

### Semi-quantitative Reverse-transcription PCR

RNA samples were extracted in spores, mature gametophytes, sporophytes, petiols, and roots. The quality and quantity of RNA were analyzed by spectrophotometry and agarose gel electrophoresis, respectively. cDNA was synthesized from 500 ng of total RNA using a cDNA synthesis kit (TaKara). About 5% of the synthesized cDNA was used in each PCR reaction, and target transcripts were amplified with specific primers in a PCR amplification procedure using *18S RNA* (GU385474) as a control. The primers used were as follows: *DfMADS* 1st-f 5′-GAAGAAAGCCCATGATCTGTC-3′ and *DfMADS* 1st-r 5′-CAAAGTCTCCTTGACCTCCCAG-3′, as well as *18S*-f 5′-ACTGGTCGCTCCGCCCTTTCTGT-3′, and *18S*-r 5′-GTGGTGCCCTTCCGTCAAT TCCT-3′.

### Quantitative RT-PCR and Data Analysis

Total RNA was extracted from different tissues and purified using DNase I (RNase-free) (TaKaRa). cDNA was synthesized from 500 ng of total RNA using a cDNA synthesis kit (TaKara). RT-qPCR experiments were performed on an ABI Prism 7500 sequence detector using a SYBR Green kit (SYBR ExScrip RT-PCR Kit; Takara, Japan). Data were subsequently analyzed with ABI Prism 7500 system software (Applied Biosystems Co., Ltd., USA). Relative quantification values and standard deviations were calculated using a standard curve.

Three replicates for each RNA sample were included in all experiments. As a control, parallel amplification reactions were performed with primers specific for the reference gene *18s RNA*. The PCR primers used were as follows: *DfMADS1*-f 5′-GCGTATTAGAGCAAAGAAGGAAGAG-3′ and *DfMADS*1-r 5′-CCAGGTTTCGGGAGACAGTTAC-3′, as well as *18S RNA*-f 5′-GCTTTCGCAGTA GTTCGTCTTTC-3′ and *18S RNA*-r 5′-TGGTCCTATTATGTTGGTCTTCGG-3′.

### Sequence and Cladistic Analyses

Sequence analysis of MADS-box proteins was performed using Clustal X software [20]. Bootstrap analyses were conducted with 100 replicates using the PHYLIP 3.69 program based on the neighbor-joining method to obtain optimal trees [21].

The MADS-box proteins analyzed in this study included CRM1 (CAA69276), CRM3 (CAA69407), CRM4 (CAA69408), CRM5 (CAA69409), CRM6 (CAA69410), CRM7 (CAA69411), CMADS1 (AAC24492), CMADS2 (AAC24493), CMADS3 (AAC24319), CMADS4 (AAC24320), CMADS6 (AAC24325), OPM1 (CAA69412), DfMADS1 (ADC55529), CerMADS1 (BAA25245), CerMADS2 (BAA25246), CerMADS3 (BAA25247), FBP3 (CAA50549), PMADS2 (CAA49568), SLM2 (CAA56656), PrMADS4 (AAB58907), PrMADS5 (AAB80808), PrMADS6 (AAB80809), PrMADS9 (AAB80806), AGL12 (AAC49085), SbMADS2 (AAB50181), DAL2 (CAA55867), ZAG2 (CAA56504), AGL11 (AAC49080), ZAG1 (AAA02933), AGL1 (AEE79831) and AGAMOUS (AEE84112).

## Results

### Sequence Analysis of Full-length cDNA

Two *MADS* gene fragments were successfully amplified by 5′- and 3′-RACE from young plants of *D. fragrans*. A cDNA clone with the expected size (approximately 1.0 kbp), named *DfMADS*1, was finally identified by PCR in *D. fragrans.* The *DfMADS*1 cDNA was 973 bp long and contained an ORF of 243 amino acid residues with a 75 bp 5′-UTR and a 169 bp 3′-UTR. The sequence data were submitted to GenBank under accession number GU835475.

**Figure 1:**
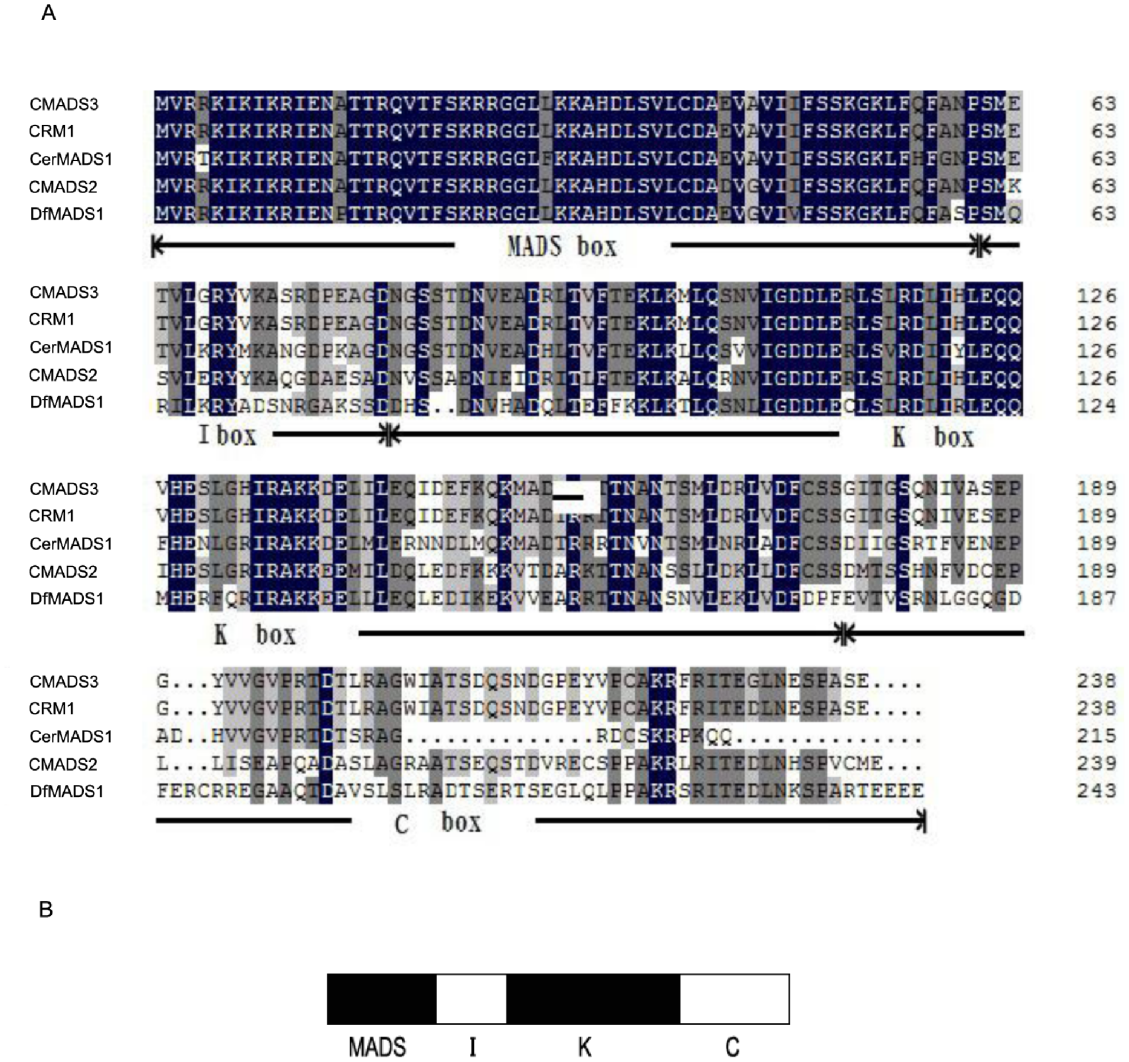
Alignment of the deduced amino acid sequences of the DfMADS1 protein with those of other related MADS-box proteins of ferns (A). The MADS and K domains are underlined. Schematic of the MIKC-DfMADS protein in *D. fragrans* (B).

Based on the deduced amino acid sequence, DfMADS1 showed high homology with several MADS-box proteins in other ferns. DfMADS1 respectively shared 63%, 63%, 61%, and 61% identity with CMADS2, CMADS3, CRM1, and CerMADS1 of *C. richardii*. Multiple sequence alignments indicated that DfMADS1 protein has a typical MIKC-type domain structure consisting of a MADS domain, a K domain, a short I region, and a C-terminal region. The highly conserved MADS domain spans amino acids from 1 to 61, and the K domain spans amino acids from 81 to 174 (Fig. 1).

### Phylogenetic Relationships of the MADS Proteins in Ferns

To better understand the relationships of *MADS-box* genes among ferns and seed plants, phylogenetic analysis was performed using the deduced amino acid sequences of reported MIKC-type *MADS-box* genes from *C. richardii*, *C. pteridoides, D. fragrans*, and *Ophioglossum pedunculosum.* Other MADS-box genes may be obtained from *Pinus radiata*, *Picea abies*, *Arabidopsis thaliana*, *Petunia × hybrida*, *Silene latifolia*, *Sorghum bicolor*, and *Zea mays*.

**Figure 2:**
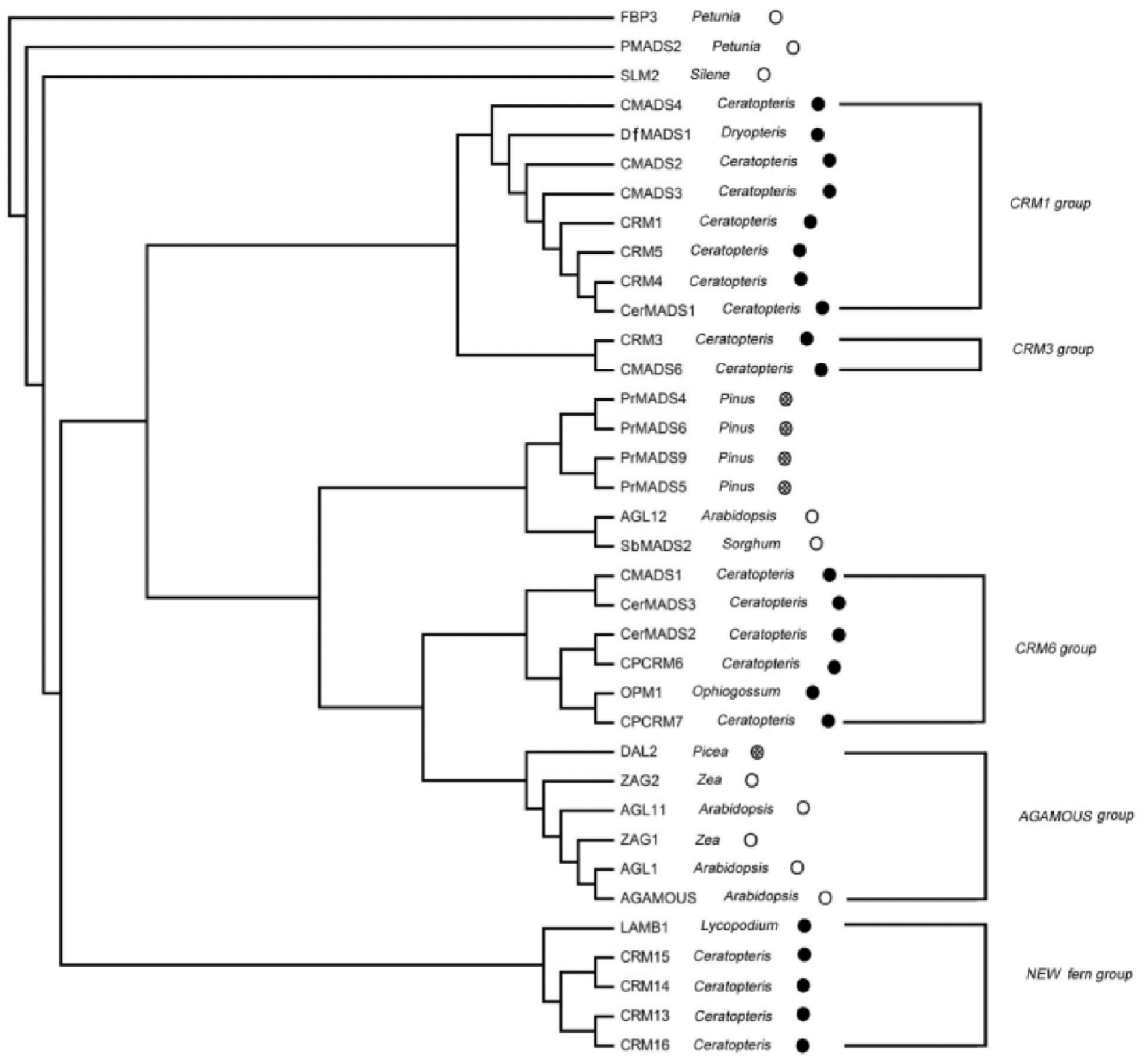
Neighbor-joining-based phylogenetic tree of fern MADS-box proteins. The tree is unrooted. The symbols after the gene names indicate spermatophytes (open circles), gymnosperms (hatched circles), and pteridophytes (solid circles). DfMADS1 was grouped into the CRM1-like subfamily.

The phylogenetic tree of the MADS-box proteins showed that CMADS1, CerMADS3, CRM7, OPM1, CRM6, and CerMADS2 can be grouped into the CRM6-like group, whereas CRM3 and CMADS6 belong to the CRM3-like group. Furthermore, the DfMADS1 protein, and CMADS2, CMADS4, CRM1, CRM4, and CRM5 formed a clade, which suggests that DfMADS1 probably belongs to the CRM1-like subfamily (Fig. 2).

### Expression Analysis of the *DfMADS1* Gene

**Figure 3:**
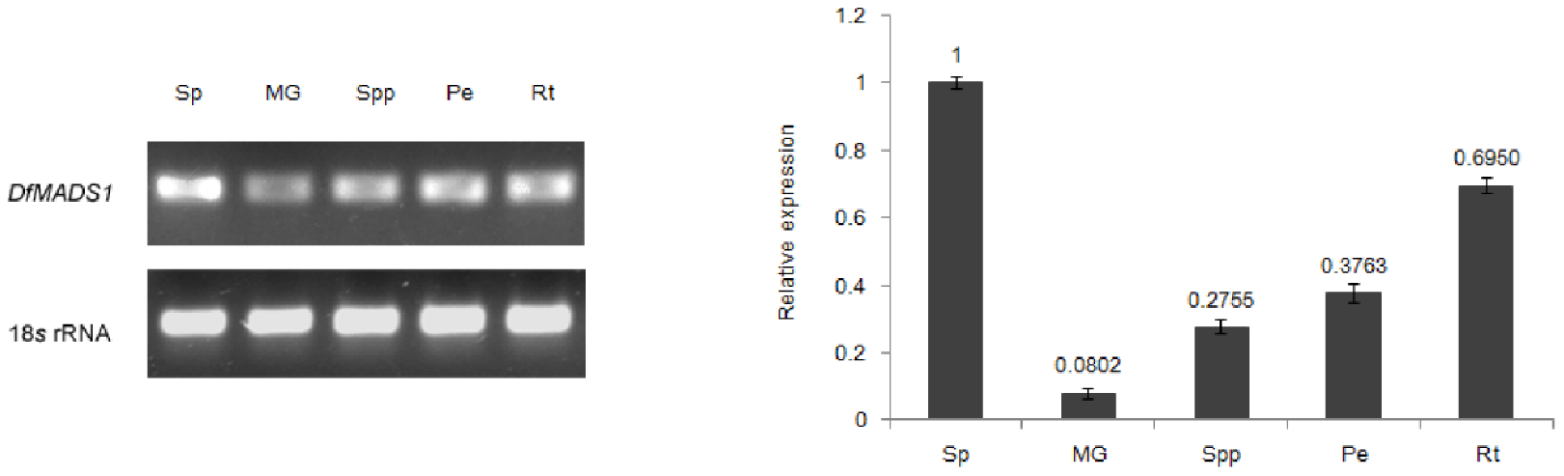
Expressions of *DfMADS1* gene in various tissues and organs of *D. fragrans*. Total RNA from spores (Sp), mature gametophytes (MG), sporophytes (Spp), petiols (Pe), and roots (Rt) was used for semi-quantitative PCR and qRT-PCR analyses. Relative mRNA levels of the *DfMADS1* gene were normalized against 18S rRNA. Error bars indicate +/− standard errors from three biological replicates.

Semi-quantitative RT-PCR was performed to explore the expression of *DfMADS1* gene in different organs of *D. fragrans* at different developmental stages. The expression of *18S rRNA* in leaves was used as a calibration standard. *DfMADS1* was expressed in spores, mature gametophytes, sporophytes, petiols, and roots, as shown in Fig. 3. In different developmental stages, the expression of *DfMADS1* gene was relatively low in the mature gametophyte of *D. fragrans*; the highest expression was observed in spores.

Quantitative RT-PCR was subsequently applied to quantify the transcript levels of the *MADS-box* gene among samples. (Fig. 3). Consistent with the semi-quantitative RT-PCR results, different expression levels of the *DfMADS1* gene were observed in different tissues of *D. fragrans*. Compared with that in mature spores, the expression level of the *DfMADS1* gene was relatively low in roots, gametophytes, sporophyte leaves, and petioles.

**Figure 4:**
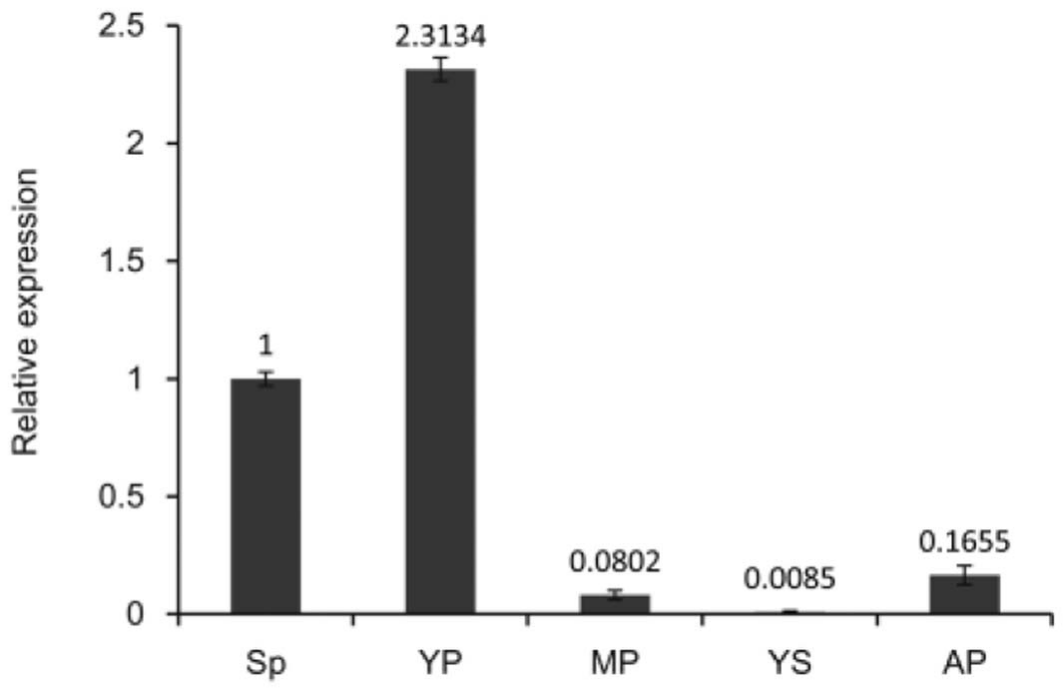
Expression of *DfMADS1* gene in the gametophytes of *D. fragrans*. Total RNA from the spore (Sp), young prothallus (YP), mature prothallus (MP), young sporophyte (YS), and aborted prothallus (AP) was used for qRT-PCR analysis. Error bars indicate +/− standard errors from three biological replicates.

Because *DfMADS1* showed the highest expression in spores, we further compared its expression in gametophytes and sporophytes. RNAs were extracted from gametophytes at different developmental stages. qRT-PCR analysis indicated that the transcript of *DfMADS1* increased by approximately 2.5-fold after spore germination (Fig. 4). When the mature prothallus was formed, *DfMADS1* transcripts decreased sharply (over 10-fold decrease). The transcript level of *DfMADS1* remained at low levels during the development of young sporophytes. At this stage, archegonia are aborted when the egg is not fertilized. Specify in which developmental stage this increase in transcript level was observed. but remained significantly lower (∼5-fold lower) than that in spores.

**Figure 5:**
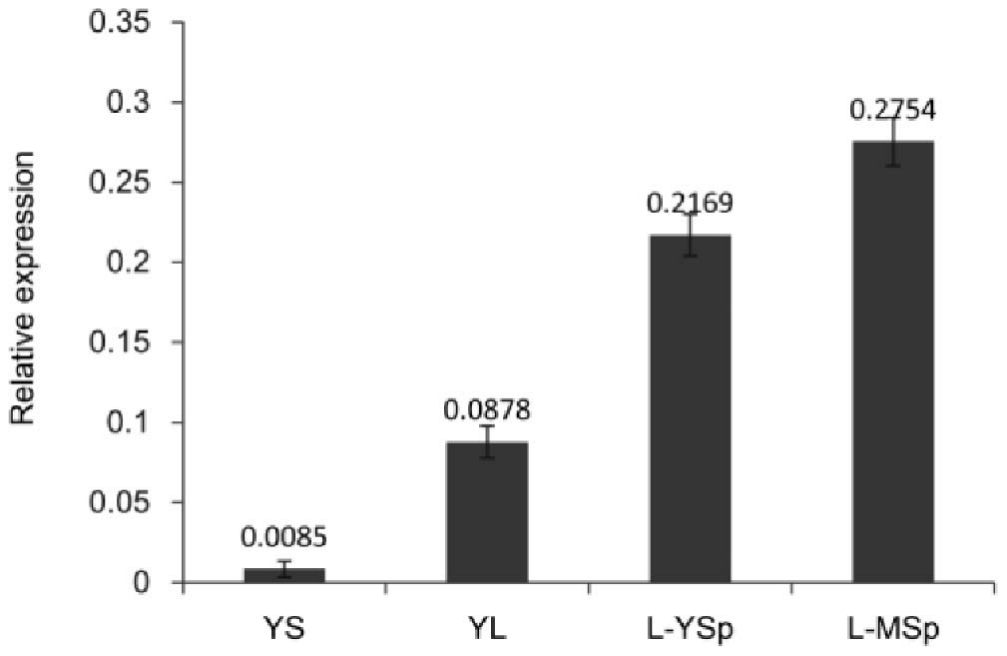
Expression of *DfMADS1* gene in sporophytes of *D. fragrans*. Total RNA from young sporophytes (YS), young leaves (YL), leaf-young sporangium (L-YSp), and leaf-mature sporangium (L-MSp) was used for qRT-PCR analysis. Error bars indicate +/− standard errors from three biological replicates.

We further examined the expression of the *DfMADS*1 gene in sporophytes. The transcript levels of the *DfMADS1* gene during all sporophyte stages were lower than the transcript levels of the *DfMADS1* gene in mature spores (Fig. 5). DfMADS1 levels were lowest (∼100-fold lower) at the young sporophyte stage compared with other developmental stages. The expression level of *DfMADS1* then increased gradually in subsequent sporophyte stages.

## Discussion

A new *MADS-box* gene (*DfMADS1*) was isolated from *D. fragrans.* Sequence analysis revealed that the deduced amino acid sequences of *DfMADS1* shared high homology with the MADS box proteins of other pteridophytes. A highly conserved MADS-box domain and relatively conserved K domain were observed in *D. fragrans* and other pteridophytes (Fig. 1). These results indicate that DfMADS1 is a typical MIKC-type MADS-box protein.

The temporal and spatial expression patterns of five *MADS* genes (*CMADS*1, 2, 3, 4, and 6) in *C. richardii* have been analyzed by Northern blot and in situ hybridizations. *CMADS*1, 2, 3, and 4 are expressed similarly in gametophytic and sporophytic tissues, whereas *CMADS6* is expressed only in hermaphroditic gametophytes [15]. Similar to the former four genes of *C. richardii*, the *DfMADS*1 gene was expressed in both gametophytes and sporophytes of *D. fragrans*, as determined by semi-quantitative and quantitative RT-PCR analyses.

We further found that the expression levels of *DfMADS*1 vary significantly during spore germination and reproductive development. These results suggest that the expression of DfMADS1 is closely associated with spore germination and reproductive development in *D. fragrans.*

Using gene chip technology, over 14,000 genes of *C. richardi* were found to be involved in gene expression and protein metabolism in the spore germination process [22]. The expression level of the *DfMADS1* gene during spore germination was very high, which indicates that the *DfMADS1* gene in *D. fragrans* may be related to gene regulation during spore germination.

In flowering plants, some *MADS* genes are expressed in both reproductive and vegetative organs, whereas other *MADS* genes are expressed in specific floral organ primordia as homeotic selector genes [23]. The *DfMADS*1 gene in *D. fragrans* was found to be expressed in both reproductive and vegetative organs in our study, similar to four of the five *MADS* box genes in *C. richardii.* These findings suggest that the ancestral *MADS* gene(s) are widely expressed in developing organs of plant. However, the *LAMB1* gene, a MADS-box gene in *L. annotinum*, is expressed exclusively in the reproductive structure and strobilus during sporogenesis [24]. The expression of the *DfMADS*1 gene in our study showed significant changes during spore germination and reproductive organ development, which suggests that the *MADS* gene may have an important role in the development or evolution of floral organs.

Prior to the year 2000, *MADS-box* genes obtained in *C. richardii* were divergent and fell into three groups, namely, CRM1-like, CRM3-like, and CRM6-like [15]. *LAMB1* of *Lycopodium annotinum* does not belong to any *MADS-box* subfamily in the three fern groups [24]. In 2012, four MADS-box genes of *C. richardii* (CRM13, CRM14, CRM15, CRM16) were cloned to study the evolution of MIKC* MADS-box genes [25]. Based on the works of Hasebe et al. and Kwantes et al., our phylogenetic analysis showed that *LAMB1* and four new MADS-genes may be grouped into a new fern MADS group and that the *DfMADS1* gene obtained in our study belongs to the CRM1-like subfamily (Fig. 2).

To date, only three or four *MADS* gene subfamilies have been found in ferns, much fewer than the *MADS-box* subfamilies in angiosperms [1], [26], [27]. This finding is consistent with the hypothesis that duplication of the *MADS* genes and their subsequent divergence is related to the cooption of *MADS* genes as homeotic selector genes and the increase in the complexity of reproductive organs observed in angiosperms.

Pteridophytes, as an appropriate and special research material, lie in the middle of the evolutionary tree of vascular plants, representing a transitional taxonomic group between angiosperms and bryophytes. Both the gametophytes and sporophytes of pteridophytes can survive independently, it has apparent alternation of generations, which is a continuous and systemic development process. MADS box genes are important regulators of developmental control, and ferns have a unique evolutionary position; thus, pteridophytes are an important research material in the evolution of *MADS box* genes. We believe that the function of MADS box genes in ferns deserves further studies.
